# Integrated medical school ultrasound: development of an ultrasound vertical curriculum

**DOI:** 10.1186/2036-7902-5-6

**Published:** 2013-07-02

**Authors:** David P Bahner, Eric J Adkins, Daralee Hughes, Michael Barrie, Creagh T Boulger, Nelson A Royall

**Affiliations:** 1 Department of Emergency Medicine, The Ohio State University College of Medicine, 750 Prior Hall, 376 W 10th Avenue, Columbus, OH 43210, USA; 2 Division of Pulmonary, Allergy, Critical Care, and Sleep Medicine, Department of Internal Medicine, The Ohio State University College of Medicine, 750 Prior Hall, 376 W 10th Avenue, Columbus, OH 43210, USA; 3 Department of Surgery, Orlando Health, Orlando, FL 32806, USA; 4 University of Central Florida College of Medicine, Orlando, FL 32827, USA

**Keywords:** Curriculum, Focused ultrasound, Medical education, Ultrasonography, Undergraduate medical education

## Abstract

**Background:**

Physician-performed focused ultrasonography is a rapidly growing field with numerous clinical applications. Focused ultrasound is a clinically useful tool with relevant applications across most specialties. Ultrasound technology has outpaced the education, necessitating an early introduction to the technology within the medical education system. There are many challenges to integrating ultrasound into medical education including identifying appropriately trained faculty, access to adequate resources, and appropriate integration into existing medical education curricula. As focused ultrasonography increasingly penetrates academic and community practices, access to ultrasound equipment and trained faculty is improving. However, there has remained the major challenge of determining at which level is integrating ultrasound training within the medical training paradigm most appropriate.

**Methods:**

The Ohio State University College of Medicine has developed a novel vertical curriculum for focused ultrasonography which is concordant with the 4-year medical school curriculum. Given current evidenced-based practices, a curriculum was developed which provides medical students an exposure in focused ultrasonography. The curriculum utilizes focused ultrasonography as a teaching aid for students to gain a more thorough understanding of basic and clinical science within the medical school curriculum. The objectives of the course are to develop student understanding in indications for use, acquisition of images, interpretation of an ultrasound examination, and appropriate decision-making of ultrasound findings.

**Results:**

Preliminary data indicate that a vertical ultrasound curriculum is a feasible and effective means of teaching focused ultrasonography. The foreseeable limitations include faculty skill level and training, initial cost of equipment, and incorporating additional information into an already saturated medical school curriculum.

**Conclusions:**

Focused ultrasonography is an evolving concept in medicine. It has been shown to improve education and patient care. The indications for and implementation of focused ultrasound is rapidly expanding in all levels of medicine. The ideal method for teaching ultrasound has yet to be established. The vertical curriculum in ultrasound at The Ohio State University College of Medicine is a novel evidenced-based training regimen at the medical school level which integrates ultrasound training into medical education and serves as a model for future integrated ultrasound curricula.

## Background

Physician-performed focused ultrasonography represents a novel approach to patient evaluation and treatment in the acute and chronic settings [1]. Ultrasound has been utilized for decades by traditional imaging specialists (radiologists, cardiologists, and obstetric-gynecologists) within the comprehensive ultrasound paradigm, in which a sonographer performs the examination followed by a physician evaluating the images. The recent development of portable and handheld ultrasound machines has led to a rapid growth in an emerging field called focused ultrasonography [2]. Unlike traditional imaging modalities, focused ultrasound is available as a bedside tool which can be both performed and interpreted instantly by the physician. Focused ultrasound has been demonstrated to improve rapid diagnosis, clinical management, and procedural performance in multiple studies [1]. As shown in Table 1, the potential uses for focused ultrasonography are broad and cross many specialties.

**Table 1:** Currently described applications for physician-performed focused ultrasonography defined by physician specialty

**Specialty**	**Applications**
Internal medicine	Hypotension evaluation and treatment, procedural guidance
Cardiology	Cardiac evaluation, procedural guidance
Gastroenterology	Liver evaluation, intestinal obstruction evaluation, procedural guidance
Infectious disease	Soft tissue infection evaluation, procedural guidance
Nephrology	Renal evaluation, procedural guidance
Pulmonology	Pleural effusion evaluation, atelectasis evaluation, pneumothorax evaluation, procedural guidance
Rheumatology	Joint and tendon evaluation, procedural guidance
Pediatrics	Developmental abnormality evaluation, abdominal pain evaluation, procedural guidance
Emergency medicine	Hypotension evaluation, intrauterine pregnancy evaluation, abdominal aortic aneurysm evaluation, cardiac evaluation, biliary tree evaluation, urinary tract evaluation, venous thromboembolism evaluation, soft tissue and musculoskeletal evaluation and treatment, lung evaluation, ocular evaluation, procedural guidance
Obstetrics-gynecology	Intrauterine pregnancy evaluation, fetal development evaluation, ovary evaluation
Surgery	Trauma evaluation, breast and thyroid evaluation, vascular evaluation, intraoperative evaluation, procedural guidance
Anesthesia	Intraoperative monitoring, procedural guidance
Critical care	Hypotension evaluation, intravascular volume evaluation and treatment, cardiac evaluation, lung evaluation, intracranial pressure monitoring, procedural guidance
Neurology	Transcranial evaluation
Neurosurgery	Transcranial and intracranial evaluation, intraoperative guidance
Orthopedic surgery	Joint and tendon evaluation and treatment, procedural guidance
Urology	Urinary tract evaluation
Plastic surgery	Intraoperative evaluation

In recognition of the potential role of focused ultrasound within clinical practice, the American Medical Association (AMA) has affirmed that bedside focused ultrasound is within the scope of practice of appropriately trained physicians [3,4]. Governing bodies for physician specialties have also begun to define the scope of practice for physician-performed focused ultrasonography. For example, the American College of Emergency Physicians (ACEP) defined the use of ultrasonography in trauma, intrauterine pregnancy, abdominal aortic aneurysm, cardiac, biliary, urinary tract, venous thromboembolism, soft tissue, musculoskeletal, thoracic, and ocular evaluation and procedural guidance to be within the emergency physician's scope of practice [5,6]. Moreover, billing and reimbursement for focused ultrasound has been supported through the AMA Physicians' Current Procedural Terminology (CPT) 2011 codes which include CPT code modifiers for focused ultrasonography [7]. Despite supporting evidence and administrative policies for physician-performed focused ultrasonography, an educational strategy and widely accepted guidelines for competency for focused ultrasonography have yet to be established.

### Integration of ultrasound into medical education

A central issue in training physicians in ultrasonography lies in locating the time and funding for training programs. Initial attending-level programs provided evidence for the use of physician-performed focused ultrasonography, and participants demonstrated rapid skill acquisition and improved interpretation skills following these courses [8]. These courses however are generally limited to hospital-based programs or subspecialty-based educational forums which are further limited by single-session time constraints and are unable to provide longitudinal training and ongoing skill assessment.

Currently focused ultrasonography is a required component of Emergency Medicine, Obstetrics-Gynecology, Internal Medicine, and Radiology and recommended for General Surgery and Anesthesiology residency training programs [9-11]. Traditional training at the resident level was first pioneered through Radiology and Obstetrics-Gynecology, although these programs were designed to teach comprehensive ultrasonography within their respective areas of interest [12]. Other specialties such as General Surgery have developed resident training in focused ultrasonography, in particular for evaluation and management of traumatic injuries [13]. The experiences gained in these programs vary greatly, and to date many general surgeons may rely on Emergency Medicine or Radiology physicians to perform a Focused Assessment with Sonography in Trauma (FAST) examination. Perhaps the most advanced training programs in focused ultrasonography have come from Emergency Medicine, where residents are recommended to develop a proficiency in 11 different ultrasound foci [9,14]. However, these recommendations have not been accompanied by curricula, competency assessment, or a method to standardize the quality and quantity of focused ultrasound exposure across programs. This variability between specialties and even residency programs demonstrates a need for a common foundational curriculum.

### Role of ultrasound within medical school

Focused ultrasonography has the potential to be both an educational adjunct and a clinical tool for medical students [15-29]. Early analyses demonstrated that in small cohorts, medical students were able to develop the psychomotor and interpretative skills required for effective focused ultrasonography [17,20,22,25,26,29]. For example, a study at Wayne State University showed that first-year medical students were able to successfully utilize portable ultrasound to differentiate sonographic objects following six 90-min sessions covering abdominal, cardiovascular, genitourinary, and musculoskeletal applications [29]. Additional efforts have demonstrated that focused ultrasonography may be useful as an educational aid in teaching anatomy to medical students [15,16,18,19,27]. A study from the Mayo Clinic demonstrated that fourth-year medical students who used focused echocardiography to aid in the understanding of cardiovascular anatomy had high satisfaction rates [15]. Further, the use of focused ultrasonography among medical school students has been shown to potentially aid in the development of physical examination skill acquisition [19,28]. In a study from the University of Chicago, fourth-year medical students used focused echocardiography in cardiac evaluation with subsequent improved detection of cardiac conditions and higher accuracy in cardiac auscultation skills [19].

Medical students have been shown to adequately develop focused ultrasound skills for clinical practice through multiple small studies [21,28,30]. Despite the demonstrated benefits, there have been only limited efforts to develop a comprehensive and organized curriculum utilizing focused ultrasonography in medical school [28,31]. The need for a vertical curriculum which incorporates ultrasound education into an existing medical school curriculum was the basis for development of an ultrasound vertical curriculum at The Ohio State University College of Medicine (OSU COM).

## Methods

The purpose of the ultrasound vertical curriculum was to develop medical student proficiency in focused ultrasonography that would allow graduates to (1) identify appropriate uses of focused ultrasonography, (2) acquire real-time ultrasound images and video, (3) interpret ultrasound findings, and (4) utilize ultrasound findings in clinical decision-making. A curriculum was developed based upon three learner theory model core components: cognitive, behavioral, and constructive. Due to curriculum time constraints, the decision was made to emphasize cognitive and behavioral learning components within mandatory courses and include constructive components predominately within the elective or selective courses. Table 2 shows the integration of vertical ultrasound curriculum components within the current medical school curriculum.

**Table 2:** An overview of four-year integrated vertical ultrasound curriculum developed at The Ohio State University College

**Medical school year**	**Components**			
Medical School Year 1	3 Months	Gross Anatomy and Laboratory	Musculoskeletal Anatomy Ultrasound	
			Thorax, Abdomen and Pelvis Anatomy Ultrasound	
			Head and Neck Anatomy Ultrasound	
	7 Months	Basic Science Curriculum	Introduction to Focused Ultrasound Elective^a^	
	2 Months	Vacation		
Medical School Year 2	8 Months	Basic Science Curriculum	Basic Course in Focused Ultrasound Protocols Elective^a^	Ultrasound Model Pool Elective^a^
	2 Weeks	Introduction to Clinical Medicine	Ultrasound-Guided Vascular Access	
	2 Months	Vacation		
Medical School Year 3^b^	8 Weeks	Internal Medicine	Specialty-Based Hands-On Ultrasound Experience	
	8 Weeks	Pediatrics		
	8 Weeks	Family Medicine		
	8 Weeks	General Surgery		
	8 Weeks	Neurology/Psychiatry		
	6 Weeks	Obstetrics-Gynecology		
	2 Weeks	Clinical Topics	Core Focused Ultrasound Protocols	
Medical School Year 4	1 Month	Emergency Medicine Rotation	Emergency Focused Ultrasound	Advanced Course in Focused Ultrasound Elective^a^
	1 Month	Ambulatory Medicine Rotation	Specialty-Based Hands-On Ultrasound Experience	
	1 Month	Chronic Care Rotation		
	1 Month	Sub-Internship Rotation		
	7 Months	Elective Rotations		

^a^An elective course which is not mandatory for students; ^b^only selected specialties are participated.

### Objectives of an integrated curriculum

The curriculum was constructed to accomplish objectives within focused ultrasonography which are complimentary to the larger medical school curriculum. Given the predominance of basic science and clinical skills taught during the first 2 years of the curriculum, emphasis on ultrasound basic science and image acquisition was chosen. Within the clinical years, an emphasis on ultrasound indications and interpretation was selected.

In the first medical school year, the curriculum covered basic ultrasound physics including ultrasound wave production, propagation within media, and image production. Also, students learned how to utilize a portable ultrasound machine to acquire ultrasound images and identify basic anatomy given respective acoustic properties. In the second medical school year, students learned advanced ultrasound physics including variations in ultrasound transducer wave production, artifact production, and thermo-mechanical heat production with a basic understanding of associated relative risks. Following the second year, students were able to understand image acquisition limitations and methods to overcome challenges to image acquisition as well as utilize ultrasound during basic procedural guidance. In the third medical school year, students learned to identify general indications for specific focused ultrasound examinations and the proper timeline in the clinical scenario for their use as well as gaining a deeper understanding of ultrasound for procedural guidance. In the fourth medical school year, students were able to identify limitations of focused ultrasonography and identify the sensitivity and specificity for focused ultrasonography for basic ultrasound protocols as well as understand risks and benefits associated with focused ultrasonography in clinical scenarios. Students were able to utilize focused ultrasonography at least within the scope of practice of their chosen specialty prior to graduation.

### Facilities and faculty

The OSU COM is a single-campus university-based college with a university-based medical center on campus. The OSU COM is a 4-year medical school with 2,779 full-time faculty and approximately 863 students. The college curriculum is a traditional structure with two preclinical years of basic science and integrated clinical exposures followed by two clinical years. The preclinical course structure is an organ-based structure and contains longitudinal clinical mentorship for instruction. The clinical years consist of month-long rotations with mandatory rotations in Family Medicine, Internal Medicine, Pediatrics, Surgery, Obstetrics-Gynecology, Neurology, and Psychiatry during the third year and selective rotations throughout the third year with a mandatory fourth-year Emergency Medicine rotation. The medical center consists of a 1,177-bed hospital system with 1,158 full-time clinical faculty in addition to residency and fellow training programs. The medical center contains portable ultrasound machines available for physician use within the Emergency Department, Surgical and Medical Intensive Care Units, Radiology Department, and medical and surgical patient floors.

The OSU COM contains a staffed simulation and training center (OSU COM Clinical Skills and Education Assessment Center) containing lecture rooms, simulation labs, and clinical rooms shared by the college and medical center. The clinical skill center simulation equipment includes ultrasound simulation models such as vascular phantoms for ultrasound-guided vascular access and pelvic phantoms for obstetrics and gynecology ultrasound examinations. Additionally, the center contains a dedicated ultrasound practical scanning center with four portable ultrasound machines with image and video archival systems.

### Curriculum description

#### Medical school year 1

Numerous gross anatomy adjuncts exist to aid understanding anatomical relationships and serve as an introduction to image comprehension. Unlike computed tomography, magnetic resonance imaging, or X-ray, ultrasound allows for hands-on exploration of anatomy. Medical students are provided 2-h-long lectures on basic ultrasound principles. The lectures review ultrasound wave production, propagation in tissue, and image production in addition to portable ultrasound machine knobology, transducers, and image acquisition techniques. Students participate in 1-h supervised hands-on sessions during each anatomy region. Students obtain ultrasound images of all required anatomical relationships for the respective anatomy region. Sessions are led by the ultrasound faculty and an anatomy professor. Individual guidance is provided by fourth-year medical students, and student volunteers serve as live models. Table 3 shows the anatomical structures and regions covered in the course. The practical anatomy examination for each anatomical region includes anatomy identification questions using ultrasound images and video.

**Table 3:** Anatomical structures acquired and identified on live models during the ultrasound component of gross anatomy

**Anatomical region**	**Anatomical structure**
Thorax and abdomen	Cardiac chambers (right and left ventricles, right and left atria)
	Pericardium
	Inferior vena cava (intra-abdominal and intrathoracic)
	Diaphragmatic caval hiatus
	Cavo-atrial junction
	Main portal vein
	Intrahepatic portal veins (right and left)
	Hepatic veins (right, middle, and left)
	Gallbladder
	Kidney (right and left)
	Spleen
	Hepatorenal recess (Morrison's pouch)
	Aorta (intra-abdominal)
	Celiac trunk, splenic artery, common hepatic artery, left gastric artery
	Superior mesenteric artery
	Inferior mesenteric artery
	Renal artery (right and left)
	Common iliac arteries (right and left)
Upper extremities	Supraspinatus tendon
	Subscapularis tendon
	Biceps brachii tendon
	Flexor digitorum profundus
	Flexor digitorum superficialis
	Flexor pollicis longus
	Median nerve
Lower extremities	Quadriceps muscle belly and central tendon
	Patella
	Patellar ligament
Head and neck	Trachea
	Thyroid gland
	Thyroid cartilage
	Internal jugular vein
	Common carotid artery
	Sternocleidomastoid

After gross anatomy, a supplemental course is available for approximately 80 students to increase hands-on time and improve basic ultrasound education. The course utilizes the evaluation of a hypotensive patient with focused ultrasound as the main construct, with the established Trinity protocol as the primary assessment method [32]. Monthly lectures and hands-on sessions review the basic concepts covered during the gross anatomy ultrasound course and further explain concepts such as ultrasound wave production, propagation in media such as tissue, air, or fluid, and thermo-mechanical effects. The lectures explain the I-AIM model (indication, acquisition, interpretation, and medical decision-making) to students as a guide for utilizing focused ultrasonography [33]. Students are taught to recognize ultrasonographic findings such as pericardial effusions, cardiac motility disorders, abdominal free fluid, and aortic aneurysms. A post-course examination consisting of a practical examination on live student models and a written image identification examination is held to assess proficiency.

#### Medical school year 2

In preparation for the transition to clinical applications of preclinical training, medical students at OSU COM are exposed to a 2-week hands-on clinical skill review session in conjunction with a clinical reasoning course. An introduction to ultrasound-guided and ultrasound-assisted procedures is provided during the course by performing ultrasound-guided long- and short-axis peripheral and central vascular cannulation. Students are required to successfully differentiate venous from arterial vasculature using B-mode, compression, Doppler, and color ultrasonography. Online lectures through the EMSono site, an online learning management website, provide students with introductory didactics on basics in ultrasound as an adjunct to hands-on training [34]. Post-didactic lecture quizzes are provided to ensure adequate understanding prior to completion of the hands-on sessions. A 1-h review of the procedural indications for ultrasound guidance and assistance as well as potential challenges in use such as image artifact production, site contamination, and equipment requirements is provided.

In coordination with organ-based topics during the second pre-clinical medical school year, an elective course teaching focused ultrasound examination of relevant organ systems is available for approximately 40 students. The primary objectives of the longitudinal course are to provide increased exposure and review of basic ultrasound principles, additional focused ultrasound protocols, and hands-on experience with portable ultrasound machines. Basic ultrasound principles are presented, including machine operation such as patient identification, image orientation and labeling standards, and production of images and video using secondary and tertiary functions such as color or power Doppler, measurements, and labels. Ultrasound protocols and organ systems covered through the course are shown in Table 4. Additional required interactive online lectures are provided through the online resource EMSono, reviewing the topics covered in didactic lectures. Students record a log of ultrasound images and video supporting each ultrasound protocol completed and must perform at least five ultrasound examinations in each focus to meet a minimum of 25 examinations. Participation as an ultrasound model is an additional educational component used for the course to expose students to patient-focused issues such as patient positioning, probe pressure limitations, and anatomical limitations. Students were required to complete at least 10 hours of live ultrasound modeling as part of the course.

**Table 4:** Organ-systems and focused ultrasound protocols covered during elective longitudinal ultrasound course for second-year medical students

**Topic**	**Focused ultrasound protocol**	**Ultrasound images/video**
Abdomen	FAST	Perihepatic
		Perisplenic
		Pericardial (subxiphoid)
		Recto-uterine/recto-vesical
	Abdominal aortic aneurysm	Celiac trunk
		Superior mesenteric artery
		Renal arteries
		Common iliac arteries
	Inferior vena cava collapsibility index	Suprahepatic IVC during expiration
		Suprahepatic IVC during inspiration
Thoracic	Pneumothorax	Pleural sliding
		M-mode pleural sliding
	Cardiac	Parasternal long axis
		Parasternal short axis (ventricle apex, middle, and base)
		Apical
		Subxiphoid
Procedural	Vascular access	Differentiation of artery and vein
		Needle vessel entry in short axis
		Needle vessel entry in long axis

#### Medical school year 3

In the mandatory core clinical rotations during the third medical school year, OSU COM students complete an adjunct course focused on enhancing their understanding of ultrasound indications and image interpretation. The third year course consists of online interactive lectures, didactic lectures, and hands-on sessions. Previously described ultrasound protocols include the Trinity hypotensive examination, FAST, cardiac evaluation during ACLS, transvaginal pregnancy evaluation, and ultrasound-guided vascular access. Students complete an online interactive lecture through the EMSono resource prior to the course. A 2-h didactic lecture covers specific indications for focused ultrasonography and ultrasound image interpretation techniques and sample images [34]. Hands-on sessions then have students demonstrate focused ultrasonography in the required protocols, with an emphasis on evaluation of a clinical scenario and ultrasound image and video interpretation.

#### Medical school year 4

In conjunction with the mandatory Emergency Medicine rotation, fourth-year students are taught to develop practice management algorithms which incorporate focused ultrasonography as part of evidenced-based practices. Students complete online lectures in focused ultrasonography using the EMSono resource prior to the initial focused ultrasound session [34]. A 2-h didactic lecture on evidenced-based evaluation of a hypotensive patient is given, which includes a review of focused ultrasonography principles from the previous coursework such as ultrasound indications, acquisition, and interpretation. Students then complete a faculty-led hands-on session where students demonstrate an evaluation of a hypotensive patient, including the previously described Trinity hypotensive protocol, transvaginal evaluation for pregnancy, and placement of central venous catheters under ultrasound guidance. As part of the rotation, students demonstrate focused ultrasound examinations on Emergency Department patients in appropriate clinical scenarios under faculty guidance, consistent with the current ACEP ultrasound scope of practice guidelines [6].

Students entering specialties with demonstrated focused ultrasonography applications are able to enroll in an elective course in advanced ultrasound training. The previously described course holds monthly didactic lectures, hands-on sessions, and journal club sessions on a specific focused ultrasound topic. Enrollment in the course is limited to approximately 40 students per year. Didactic sessions are 2-h lectures from specialty-specific faculty discussing the indications, contraindications, acquisition techniques, and examination interpretation and management implications for the topic. Hands-on sessions are proctored by the ultrasound faculty with assistance from monthly faculty using volunteer students for practice scanning models. Journal club sessions use faculty-selected journal articles on the topic with student-led presentation and analysis of the articles. Students participate in practical scanning experiences during weekly Intensive Care Unit and Emergency Department ultrasound rounds. Students are evaluated through monthly quizzes and final examinations with mandatory requirements for logged ultrasound examinations in each ultrasound focus. Furthermore, assigned lectures and readings are provided through the online resource EMSono and previously developed YouTube presentations from OSU COM faculty.

### Ethical approval

Ethical approval was obtained from The Ohio State University Office of Responsible Research Practices and Institutional Review Board. The study was deemed exempt research and a waiver of informed consent was approved by the board for the report.

## Results and discussion

The major challenge to technology-focused curricular components generally lies with funding. Specific to focused ultrasonography, start-up costs include obtaining portable or handheld ultrasound machines and associated maintenance costs. Current models for portable ultrasound machines can range up to US$60,000 per machine including service agreements and appropriate transducers. Handheld ultrasound machines may represent a drastically less expensive, although currently limited technologically option, with some machines ranging near US$10,000 including service agreements. To limit equipment costs, this program utilized shared equipment with the medical center and relies upon clinical ultrasound machines for a large portion of the educational experience. Faculty time requirements were limited by designating a single faculty member as the ultrasound program director. Additional OSU COM faculty from departments with established ultrasound practices and experience were used to further enhance the program such as Emergency Medicine, Obstetrics and Gynecology, Radiology, Surgery, and Internal Medicine.

An additional issue facing new curricula is student time constraints and integration into existing teaching methodologies. Ultrasound has proven benefit as a teaching aid in anatomy, physiology, and clinical diagnosis and treatment [15,18,19,23]. Focused ultrasonography is a relatively novel teaching aid, and many faculty, even at academic centers, may not be proficient with the technology. The purpose of this program was to demonstrate that with limited faculty experienced in focused ultrasonography, a robust and vertical ultrasound curriculum can be created. Further, this curriculum has the potential to serve as a blueprint for other medical schools seeking to develop vertical ultrasound curricula. Focused ultrasound has been shown to have rapid uptake in basic skills and understanding, with learning being demonstrated in less than 1 day of lecture in some studies [8,35]. These aspects of focused ultrasound make the addition of ultrasound into pre-clinical and clinical medical student education potentially more feasible than other technologies which require large faculty teaching time and limited scope in medical education.

### Program improvement

Components of the vertical ultrasound curriculum are evaluated by students using a survey before and after each required or elective component. Additionally, online quizzes have been introduced which evaluate student background knowledge and knowledge gained from each curricular component. A standardized method for assessing focused ultrasound knowledge however does not exist. Furthermore, there is no standardized instrument to assess focused ultrasound examination performance. A previously described scale developed at The Ohio State University College of Medicine, B-QUIET, is a reliable scale for assessing focused ultrasound image quality and is used for many of the course components [36]. Further research is needed to develop these methods for student evaluation and program efficacy.

As with all technologic advances, the medical education paradigm is challenged to develop an appropriate training regimen that integrates the information necessary for future physicians. While efforts to date have focused on graduate medical education for focused ultrasound training, numerous successful attempts to utilize focused ultrasonography exist at the medical school training level. The vertical ultrasound curriculum developed at The Ohio State University College of Medicine is a novel attempt in developing a vertical curriculum for medical school-level focused ultrasound training. In a future publication, the authors hope to demonstrate the impact of the vertical ultrasound curriculum on student clinical performance and advances made in the curriculum with experience.

## Conclusions

Focused ultrasonography is an advancing evaluation and management application within medicine that assists practitioners in a rapid, noninvasive, and safe manner [1]. Evidence supporting focused ultrasonography is proliferating, and in conjunction, indications for its use are expanding. However, an ideal training program has yet to be developed despite a growing utilization of this technology. The vertical curriculum in ultrasound is an important initial effort to develop an evidenced-based training regimen at the medical school level, integrating focused ultrasonography as both a teaching adjunct and a clinical aid in training future physicians.

## Abbreviations

ACEP: American College of Emergency Physicians; AMA: American Medical Association; CPT: Current Procedural Terminology; FAST: Focused Assessment with Sonography for Trauma; OSU COM: The Ohio State University College of Medicine.

## Competing interests

The authors declare that they have no competing interests.

## Authors' contributions

DPB conceived the vertical curriculum. DPB, EJA, and CTB contributed significantly to the design and implementation of the curriculum. NAR, DH, and MB performed the acquisition of data, analysis and interpretation of data, and initial manuscript preparation. All authors performed critical manuscript revisions. All authors read and approved the final manuscript.

## Authors' information

DPB is a Professor of Emergency Medicine, the Director of Ultrasound in Emergency Medicine, and the Emergency Medicine Ultrasound Fellowship Director in the Department of Emergency Medicine, The Ohio State University College of Medicine. EJA is an assistant professor in the Department of Emergency Medicine with joint appointment with the Division of Pulmonary, Allergy, Critical Care, and Sleep Medicine in the Department of Internal Medicine, The Ohio State University College of Medicine. CTB is an assistant professor in the Department of Emergency Medicine, The Ohio State University College of Medicine. DH and MB are residents in the Department of Emergency Medicine, The Ohio State University College of Medicine. NAR is a resident in the Department of Surgery, Orlando Health, with joint appointment as a resident instructor at the University of Central Florida College of Medicine.
